# Neuronal Actions of Transspinal Stimulation on Locomotor Networks and Reflex Excitability During Walking in Humans With and Without Spinal Cord Injury

**DOI:** 10.3389/fnhum.2021.620414

**Published:** 2021-02-18

**Authors:** Md. Anamul Islam, Timothy S. Pulverenti, Maria Knikou

**Affiliations:** ^1^Klab4Recovery Research Laboratory, Department of Physical Therapy, College of Staten Island, The City University of New York, Staten Island, NY, United States; ^2^PhD Program in Biology and Collaborative Neuroscience Program, Graduate Center of the City University of New York and College of Staten Island, New York, NY, United States

**Keywords:** H-reflex, locomotion, neuromodulation, spinal cord injury, transspinal stimulation

## Abstract

This study investigated the neuromodulatory effects of transspinal stimulation on soleus H-reflex excitability and electromyographic (EMG) activity during stepping in humans with and without spinal cord injury (SCI). Thirteen able-bodied adults and 5 individuals with SCI participated in the study. EMG activity from both legs was determined for steps without, during, and after a single-pulse or pulse train transspinal stimulation delivered during stepping randomly at different phases of the step cycle. The soleus H-reflex was recorded in both subject groups under control conditions and following single-pulse transspinal stimulation at an individualized exactly similar positive and negative conditioning-test interval. The EMG activity was decreased in both subject groups at the steps during transspinal stimulation, while intralimb and interlimb coordination were altered only in SCI subjects. At the steps immediately after transspinal stimulation, the physiological phase-dependent EMG modulation pattern remained unaffected in able-bodied subjects. The conditioned soleus H-reflex was depressed throughout the step cycle in both subject groups. Transspinal stimulation modulated depolarization of motoneurons over multiple segments, limb coordination, and soleus H-reflex excitability during assisted stepping. The soleus H-reflex depression may be the result of complex spinal inhibitory interneuronal circuits activated by transspinal stimulation and collision between orthodromic and antidromic volleys in the peripheral mixed nerve. The soleus H-reflex depression by transspinal stimulation suggests a potential application for normalization of spinal reflex excitability after SCI.

## Introduction

Spinal cord injury (SCI) impairs the integration of neuromodulatory inputs from supraspinal centers and sensory organs (Rossignol et al., 2006; Côté et al., 2018). The complex disorganization after SCI results in pathological coordination of muscle activity during walking (Roby-Brami and Bussel, 1987; Calancie et al., 1993; Crone et al., 2003; Dietz and Sinkjaer, 2007). Exercise and stimulation based protocols can provide the necessary neuromodulation for disorganized spinal locomotor networks to generate rhythmical motor output and thus alleviate impairment of walking performance after SCI (Dobkin et al., 1995; Lünenburger et al., 2006; Gerasimenko et al., 2015b; Hofstoetter et al., 2017).

Proprioceptive reflexes refine muscle activity during locomotion and contribute to recovery after SCI (Lam and Pearson, 2002; Rossignol et al., 2006; Côté et al., 2018). Proprioceptors provide continuous feedback to spinal circuits by acting as forwarding sensory models that convey future kinematic states of the limbs, support patterned motor activity, and account partly for phase transitions during stepping (Miall and Wolpert, 1996; Dimitriou and Edin, 2010). Reflexes arising from muscle spindles, in particular, are essential to locomotor control because their large diameter afferents have widespread connections with motoneurons and several classes of interneurons linked directly to locomotion (Akay et al., 2014; Jankowska, 2015). Muscle spindle proprioceptive reflexes are uniquely important to locomotor recovery after SCI. Specifically, proprioceptive reflexes direct and maintain reorganization of spinal circuit dynamics promoting a more physiological phase-dependent reflex modulation and coordinated muscle activity (Knikou et al., 2009; Knikou, 2013a; Takeoka et al., 2014; Takeoka and Arber, 2019). Consequently, reflexes conveyed in muscle spindle afferents should be unimpeded during rehabilitation efforts. It is well established that the Hoffmann (H)-reflex is used to assess muscle spindle spinal reflex excitability (Capaday and Stein, 1987b; Knikou et al., 2009). The modulation of the H-reflex during walking and how it is affected by transspinal stimulation can provide significant information on neural interactions between transspinal stimulation, afferent inputs, and spinal monosynaptic-polysynaptic reflex circuits.

One of the most common rehabilitation strategies to repetitively provide physiological locomotor proprioceptive feedback is body weight support (BWS) assisted stepping. Albeit, BWS assisted stepping contributes to the recovery of locomotor ability, the sustained pathology of motor activity has led researchers and clinicians to combine locomotor training with transspinal stimulation to enhance excitation of spinal networks and locomotor output (Gerasimenko et al., 2015a; Minassian et al., 2016; Gad et al., 2017; Barss et al., 2020). For example, transspinal stimulation delivered at high frequencies contributes to patterned locomotor-like activity and step-like joint kinematics in individuals with and without SCI (Hofstoetter et al., 2013; Gerasimenko et al., 2015a; Minassian et al., 2016). Furthermore, computational and neurophysiological studies suggest that this motor behavior results partly from activation of dorsal root afferents that convey proprioceptive feedback. We recently demonstrated that the soleus transspinal evoked potential (TEP), soleus H-reflex, and soleus M-wave occlude or summate in the surface electromyogram (EMG) at specific times that tibial nerve and transspinal stimulation are delivered (Ladenbauer et al., 2010; Danner et al., 2011; Knikou, 2013b; Knikou and Murray, 2018).

In this study, we investigated the neuronal actions of transspinal stimulation on locomotor muscle activity and soleus H-reflex in able-bodied subjects. We also included a small population of individuals with SCI (*n* = 5) as an exploratory to the primary objective of establishing the modulatory effects of transspinal stimulation on soleus H-reflex during walking in able-bodied individuals. We hypothesized that the transspinal stimulation during walking retains its depressive effects on the soleus H-reflex as observed in resting individuals (Knikou, 2013b; Knikou and Murray, 2018) and that the effects are more pronounced in individuals with SCI compared to able-bodied individuals due to impaired function of spinal inhibitory networks and reduced supraspinal inputs (Mailis and Ashby, 1990; Thomas and Gorassini, 2005; Xia and Rymer, 2005; Knikou and Mummidisetty, 2011).

## Materials and Methods

### Ethical Approval and Participants

The research study received full Institutional Review Board approval (2016-1231, 2017-0261) by the City University of New York IRB committee and was conducted in compliance with the Declaration of Helsinki. Each participant signed an informed consent form before enrollment and participation in the study. Thirteen able-bodied adults (five male and eight female; 19–35 years; height 169.7 ± 5.9 cm; weight 70.3 ± 10.9 kg) without any disease or disorder and five individuals with SCI (four male and one female; 33–60 years; height 177.2 ± 5.9; weight 78.3 ± 12.2 kg) participated in the study (Table 1).

**Table 1:** Characteristics of individuals with SCI and Lokomat settings during assisted stepping.

Subject code	Age (years)	Height (cm)	Weight (kg)	Level of injury	AIS	Time post-SCI (years)	BWS (%)	LGF (%)	Medication	Speed (km/h)
1	60	170.8	91.3	C5–6	C	4	80	90	Oxybutynin 5 mg, 3/day	1.5
2	38	176.2	86.8	T11	D	9	30	65	None	2.0
3	55	174.3	71.5	C1	D	30	45	100	None	1.5
4	33	183.1	84.0	C7	B	9	80	100	None	1.5
5	33	186.8	57.7	C4	D	15	30	50	None	1.9
**Mean**	**43.8**	**177.2**	**78.3**	**–**	**–**	**13.4**	**53**	**81**	**–**	**1.7**
**SD**	**11.4**	**5.9**	**12.2**	**–**	**–**	**9.0**	**22.7**	**20.1**	**–**	**0.2**

*AIS, American spinal cord injury impairment scale; BWS, body weight support; LGF, leg guidance force; SCI, spinal cord injury. Bold text delineates mean and standard deviation values*.

### Surface EMG

Following standard skin preparation, single differential bipolar surface electrodes with a fixed inter-electrode distance of 2 cm (Motion Lab Systems Inc., Baton Rouge, LA, USA) were placed bilaterally on the soleus (SOL), medial gastrocnemius (MG), tibialis anterior (TA), and peroneus longus (PL) muscles. Surface EMG electrodes were placed over the muscle belly parallel with the underlying muscle fibers and were secured with 3M Tegaderm transparent film (3M, St. Paul, MN, USA). Surface EMG signals during walking on a motorized treadmill for control subjects or during Lokomat 6 Pro^®^ assisted stepping for SCI subjects were recorded at a sample rate of 2,000 Hz using a data acquisition card (National Instruments, Austin, TX, USA), and saved in a personal computer for off-line analysis.

### Neurophysiological Settings to Define Stimulation Parameters

First, with subjects seated the optimal stimulation sites were determined. The soleus H-reflex was evoked from the right leg based on our previous methods (Knikou, 2008, 2013a; Knikou et al., 2009, 2011). Briefly, a stainless-steel plate of 4 cm^2^ in diameter was secured proximal to the right patella. A hand-held monopolar stainless-steel head electrode was used as a probe to establish the optimal stimulation site of the posterior tibial nerve. The site corresponded to the lowest stimulus intensity at which the soleus H-reflex could be evoked without the presence of an M-wave and the shape of the M-wave was similar to that of the H-reflex. The hand-held electrode was then replaced by a pre-gelled Ag/AgCl disposable electrode (Suretrace 1800-003, ConMed, Utica, NY, USA), and was maintained under constant pressure with an athletic wrap.

The T10 spinous process was identified *via* palpation and in consolidation with anatomical landmarks. A single reusable self-adhesive cathode electrode (UniPatch EP84169, 10.2 × 5.1 cm^2^, Wabash, MN, USA) was placed longitudinally along the vertebrae equally between the left and right paravertebral sides. Due to its size, the electrode covered from T10 to L1. These levels correspond to spinal segments and segmental innervation of the ankle muscles. A pair of interconnected anode electrodes (same type as the cathode) were placed on either side of the abdominal muscles or iliac crests depending on each subject’s reported level of comfort or whether stimulation caused bladder discomfort (Murray et al., 2019). Transspinal stimulation was delivered by a constant current stimulator (DS7A for control and DS7AH for SCI subjects; Digitimer Limited, Welwyn Garden City, UK) that was triggered by Spike 2 scripts (Cambridge Electronics Design Limited, Cambridge, UK) when subjects were seated or lying supine.

**Figure 1:**
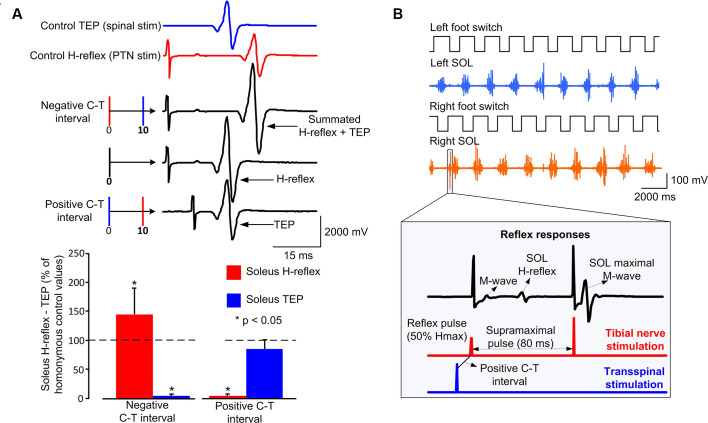
Experimental protocol: **(A)** non-rectified waveform averages of soleus transspinal evoked potential (TEP) and soleus H-reflex under control conditions, and conditioned H-reflexes following transspinal stimulation at an individualized negative or positive conditioning-test (C-T) interval. For this example, the C-T interval was 10 ms. At the negative C-T interval, the TEP and H-reflex summated on the surface EMG. At the positive C-T interval, the soleus H-reflex is completely occluded, while the TEP is not affected. The overall amplitude of conditioned soleus H-reflexes and TEPs from all subjects at a positive and negative individualized C-T interval. **(B)** Left and right foot switch signals recorded simultaneously with electromyographic (EMG) signals, and stimulation pulses delivered to the posterior tibial nerve or the thoracolumbar enlargement. At each bin of the step cycle, 60 ms after the test stimulus to the posterior tibial nerve a supramaximal stimulus was delivered to evoke a maximal M-wave that was used to normalize the associated M-wave and H-reflex. In both subject groups, the stimulating pulses were triggered based on the right footswitch signal randomly across the 16 bins of the step cycle, while a maximal M-wave was evoked after the test or conditioned H-reflex that was used in real-time to compute the percentage of the associated M-wave and H-reflex.

Subjects were then transferred to a supine position with knee and hip joints flexed at 30°, ankles supported in a neutral position, and legs maintained in midline *via* external support. The optimal position of the cathodal transspinal electrode was based on the presence of soleus transspinal evoked potential (TEP) depression in response to paired transspinal stimuli at 60 ms interstimulus interval (Murray and Knikou, 2019). The posterior tibial nerve stimulation intensity was increased progressively and the soleus maximal M-wave (Mmax) was recorded. Stimulation delivered to the posterior tibial nerve and transcutaneously to the spinal cord was set at intensities that evoked similar soleus H-reflex and soleus TEP peak-to-peak amplitude (Figure 1A), that both ranged from 20 to 30% of the Mmax and evoked on their corresponding ascending portion of the recruitment curve. This procedure ensured that a similar number and type of alpha motoneurons were excited following transspinal and posterior tibial nerve stimulation. At these stimulation intensities, the soleus H-reflex and TEP latencies were determined and used to define the individual conditioning-test (C-T) interval. We used the resultant value from equation 1 to deliver transspinal before (positive C-T interval) or after (negative C-T interval) tibial nerve stimulation during stepping (Knikou, 2017; Murray and Knikou, 2019). Note that the timing between stimuli was the same when assembled to provide a positive or negative C-T interval. We based equation 1 on similarities of amplitude modulation during walking and latency of motor evoked potentials (MEPs) and H-reflexes in healthy humans (Knikou et al., 2011, 2013). We replaced the MEP with the H-reflex in the well-established mathematical estimation of the conduction time to the presynaptic terminals of corticospinal neurons for the first descending motor volley at the spinal cord [MEP-(C_root_ + 1.5 ms) or MEP-(T_root_ + 1.5 ms)] because the MEP has similar latency to the soleus H-reflex, and we added 1.5 ms in equation 1 to allow for synaptic transmission and conduction to the lumbar nerve root at the vertebral foramina (Taylor and Martin, 2009; Bunday and Perez, 2012). In control subjects, the mean soleus H-reflex and TEP latency were 30.09 ± 1.18 and 19.41 ± 1.3 ms, respectively. These latencies resulted in C-T intervals ranging from 8 to 11 ms (9.17 ± 0.94) across control subjects. In SCI subjects, the mean soleus H-reflex and TEP latency were 31.18 ± 0.96 and 21.41 ± 0.85 ms, respectively. The C-T intervals ranged from 8 to 10.5 ms (9.3 ± 0.87) across SCI subjects.

(1) C−T interval=HReflex latency−(TEPlatency+1.5)ms

At the positive C-T interval, depolarization of motoneurons by transspinal stimulation occurs before primary Ia afferent volleys produced by tibial nerve stimulation arrive at the spinal cord and monosynaptically depolarize triceps surae alpha motoneurons. Consistent with our recently published findings (Knikou and Murray, 2018), the soleus H-reflex was depressed at the positive C-T interval (Figure 1A). Similarly, at the negative C-T interval depolarization of motoneurons by transspinal stimulation occurs after tibial nerve stimulation induces primary Ia afferent volleys. The order of these events resulted in summation between the soleus TEP and soleus H-reflex in the surface EMG (Figure 1A), consistent with our recent reported findings (Knikou and Murray, 2018).

### Neurophysiological Recordings During Stepping

Control subjects stepped at their preferred speed of 4.5–5.1 km/h on a motorized treadmill, while SCI subjects stepped on a treadmill with the assistance of a robotic-gait orthosis (Lokomat 6 Pro^®^) at a treadmill speed ranging from 1.5 to 2 km/h. Healthy control subjects were instructed to maintain balance during stimulation, while SCI subjects were instructed to work in coordination with the exoskeleton. Control subjects stepped with no BWS. The BWS, leg-guidance force, toe strap position, and treadmill speed were individualized for each SCI subject based on their ability to step without knee buckling during the stance phase and foot-dragging during the swing phase.

During treadmill walking, the soleus H-reflex was recorded under control conditions and with a single transspinal conditioning stimulation pulse (1-ms rectangular pulse). Single-pulse conditioning transspinal stimulation during stepping was calculated by establishing the soleus TEP threshold during standing and increasing the intensity until the soleus TEPs were equivalent in amplitude to the soleus H-reflex on the ascending portion of the recruitment curve during standing. Single-pulse transspinal stimulation in control subjects was delivered at 65.3 ± 26.9 mA that was equivalent to 1.45 ± 0.14 of the TEP threshold. In SCI subjects, single pulse transspinal stimulation was delivered at 248 ± 87.06 mA that was equivalent to 1.29 ± 0.01 of soleus TEP threshold. The intensity for single pulse transspinal stimulation depended on the reported comfort and especially on the bladder comfort for people with SCI, and whether the soleus TEP was of sufficient amplitude and similar to the soleus H-reflex recorded during standing. For each subject, single-pulse transspinal conditioning stimulation was delivered at an individualized positive and negative C-T interval to the soleus H-reflex during treadmill walking.

Soleus H-reflexes were recorded during walking to establish the neurophysiological modulatory effects of transspinal stimulation on spinal reflex excitability. Soleus M-wave and H-reflex recruitment curves were first assembled during standing with BWS that was equivalent to that required during stepping with the robotic-gait orthosis for SCI subjects, and without BWS for control subjects. From the recruitment curve, the stimulation intensities that evoked H-reflexes on the ascending limb of the recruitment curve ranging from 20 to 30% of Mmax and their corresponding M-wave amplitudes were determined.

During walking, the amplitude of the M-wave and H-reflex as a percentage of the Mmax, along with the stimulation intensity that these responses are evoked relative to the recruitment curve, is one of the most important factors in human experimental studies (Crone et al., 1990). To control these parameters during walking, a supramaximal stimulus was delivered to the tibial nerve 60 ms after each H-reflex stimulus that evoked a Mmax (Figure 1B). This Mmax was used by customized LabView software to calculate in real-time the preceding stimulus M-wave and H-reflex amplitude during the experiment relative to the Mmax. Based on the M-wave amplitude and the stimulation intensities of the ascending portion of the reflex recruitment curve assembled during standing, the stimulation intensity was adjusted by a self-teaching algorithm in the customized LabVIEW software to evoke M-waves within the acceptance range of 4–8% of the Mmax (Crone et al., 1990; Simonsen and Dyhre-Poulsen, 2011; Knikou, 2013a). This experimental procedure ensured that M-wave amplitudes were similar under all experimental conditions (i.e., control and transspinal conditioning stimulation).supporting the excitation of a similar group of motoneurons and afferent fibers, while H-reflexes were evoked on the ascending limb of their recruitment curve at amplitudes where inhibition or facilitation is not contaminated by polysynaptic reflex pathways (Crone et al., 1990; Knikou, 2008).

For both subject groups, control H-reflex and transspinal conditioned H-reflex stimulation paradigms during walking were recorded at different phases of the step cycle (Knikou et al., 2009). The step cycle was determined by calculating the threshold levels of the right and left-foot switches (Motion Lab Systems, Inc., Baton Rouge, LA, USA) detecting heel contact and toe-off. Based on the heel contact and toe-off detection, the step cycle was divided into 16 equal bins. Control and conditioning stimulation were then delivered randomly in the 16 bins by the customized LabView software until a minimum of 15 control and conditioning stimulation responses were recorded at each bin. Bins 1, 9, 10, and 16 correspond approximately to heel contact, stance-to-swing transition, swing phase initiation, and swing-to-stance transition, respectively. During walking, both control and conditioned H-reflexes were recorded every 3–5 steps. For each subject, at least five control and conditioned H-reflexes were recorded randomly at each bin of the step cycle.

During stepping, transspinal stimulation was also delivered alone as a pulse train of 12 pulses at 333.3 Hz with a total duration of 33 ms randomly across the step cycle. Pulse train transspinal stimulation intensity was delivered at 0.95 multiples of soleus TEP threshold, ranged from 57 to 160 mA, and produced mild trunk extension across subjects. Pulse train transspinal stimulation did not evoke ankle TEPs or bilateral leg muscle contractions during standing or stepping because higher stimulation intensities were needed during which subjects reported great discomfort. Under all conditions, EMG activity without stimulation, EMG activity for steps during and after transspinal stimulation with a single pulse and pulse train, and soleus H-reflex modulation during control conditions and following single-pulse transspinal conditioning stimulation were recorded in a single session with ample rest periods between stimulation paradigms.

### Data Analysis

#### Locomotor EMG Analysis

EMG signals during stepping were recorded from ankle flexors and extensors from both legs. For each subject separately, the EMG signals from the steps without, during, and immediately after a single pulse or pulse train transspinal stimulation were full-wave rectified, high pass filtered at 20 Hz, and low pass filtered at 500 Hz (Knikou et al., 2009; Knikou and Mummidisetty, 2014). After full-wave rectification, linear envelopes were obtained with a 20 Hz low-pass filter, and the mean EMG amplitude across all steps was established. Also, the average root mean square (RMS) EMG amplitude for the total duration of a step cycle from each participant was calculated to establish changes in motor unit recruitment during different stimulation conditions (Farina et al., 2016).

The linear EMG envelope and RMS amplitude at each bin of the step cycle during or after transspinal stimulation was normalized to the maximal EMG obtained from stepping without stimulation (Knikou and Mummidisetty, 2014). This was performed for each subject and muscle separately. The normalized EMG envelope and RMS amplitude were grouped for each subject based on the type of the step (i.e., without, during, and after a single pulse or pulse train transspinal stimulation), and the overall EMG amplitude was estimated. Spearman’s rank test was used to establish changes in EMG amplitude modulation pattern during stepping between the EMG activity recorded with single pulse or pulse train transspinal stimulation and without stimulation. The Spearman’s rank correlation was chosen because this method indicates the direction and strength of an association between two EMG signals (De Vargas Ferreira et al., 2012; Watanabe et al., 2018). The coefficient of determination of this method (r_s_) indicates the strength and direction of the correlation between two nonlinear data sets. The effect size for the correlation coefficient was determined based on Cohen’s interpretation (Cohen, 1977).

Further, the band-pass EMG signal of each muscle from the steps with or without stimulation was transformed from the time domain to the frequency spectrum using pspectrum Matlab function (MATLAB R2019a, MathWorks, USA) to establish the mean power frequency (MPF) reflecting motor unit firing rate (Fuglsang-Frederiksen and Rønager, 1988; Arendt-Nielsen et al., 1989). The MPF was calculated from the power spectrum based on equation (2).

(2) $$MPF=\frac{\sum_{i}^{N}f_{i}P_{i}}{\sum_{i}^{N}P_{i}}$$

where *f_i_* is the frequency of the EMG power spectrum at the *i*th frequency, *P_i_* is the power of the EMG spectrum at the *i*th frequency and N is the length of the frequency window. The EMG MPF was grouped for each subject group based on the type of the step (i.e., without, during, and immediately after a single pulse or pulse train stimulation) for further analysis.

Intralimb and interlimb coordination were determined based on the distribution of non-normalized EMG activity from the rectified SOL and TA muscles of the left and right legs obtained from steps without and during transspinal stimulation. An *L*-shape pattern between the antagonistic muscles (intralimb) or between the homologous muscles from the left and right legs (interlimb) support for physiological activation patterns (Knikou and Mummidisetty, 2014). To compare the shape of the distribution between antagonistic or homologous muscles for steps during or without stimulation, a two-sample Kolmogorov-Smirnov test (2-sample KS) was performed to determine the probability of the similarity of the distribution shape between the two EMG data sets observed separately for each bin of the step cycle during single pulse transspinal stimulation. When 2-sample KS resultant D-stat values were smaller than 5%, we assumed a greater probability for similar distribution shapes between two data sets (Miller, 1956; Lehmann and D’Abrera, 1975).

#### Analysis of Reflex Responses During Stepping

The soleus H-reflex, M-wave, and Mmax were measured offline as the peak-to-peak amplitude of the non-rectified waveform evoked at each bin of the step cycle. The soleus M-wave and H-reflex were normalized to the associated Mmax evoked 60 ms after the test stimulus at each bin of the step cycle (Knikou et al., 2009; Knikou, 2013a). Control and conditioned soleus H-reflexes were accepted when the M-waves ranged from 4 to 8% of the associated Mmax. The average amplitude of at least five accepted control and conditioned H-reflexes was estimated for each bin per subject and then grouped across subjects. Normal data distribution and equality of variances were assessed with Shapiro Wilk and Levene tests, respectively. For each subject group, a two-way analysis of variance (ANOVA) at three times 16 levels (3: control and conditioned H-reflexes, 16: bins of the step cycle) was performed to establish statistically significant differences between control and conditioned H-reflexes recorded during stepping.

Last, for each bin of the step-cycle, the background EMG activity was estimated from the rectified EMG at 120 ms before tibial nerve or transspinal stimulation for the 60-ms duration. This was done separately for each muscle and recording condition. The normalized soleus H-reflex was plotted on the *y*-axis against the absolute value of the associated background EMG activity on the *x*-axis and a linear least-square regression was fitted to the data. The slope of the linear relationship, which represents the gain of the H-reflex (Capaday and Stein, 1987b; Ferris et al., 2001) were grouped based on condition, and repeated measures ANOVA was applied to the data. Significance was set at *p* < 0.05 for all tests.

## Results

### Locomotor EMG Activity During and After Transspinal Stimulation

**Figure 2:**
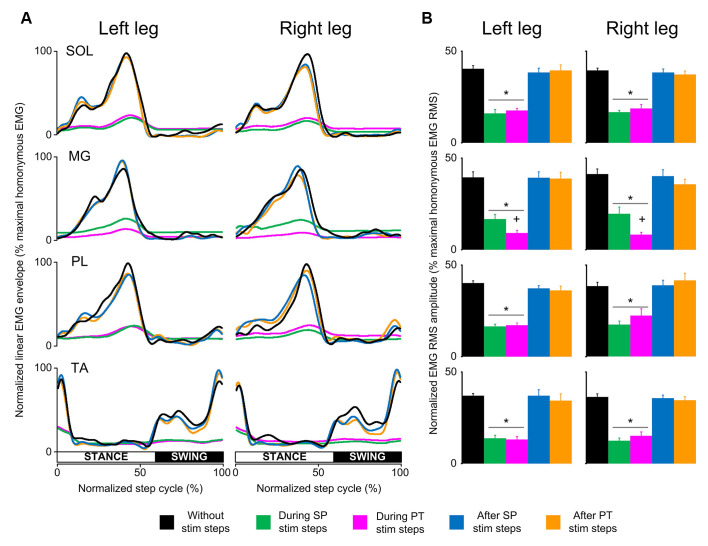
Locomotor EMG activity without, during, and after transspinal stimulation in able-bodied subjects. **(A)** Overall amplitude of normalized locomotor electromyographic (EMG) activity from soleus (SOL), medial gastrocnemius (MG), peroneus longus (PL), and tibialis anterior (TA) muscles during walking on a motorized treadmill without and during single pulse (SP; 1 ms pulse width) or pulse train (PT; 12 pulses of 33 ms total duration) transspinal stimulation. **(B)** Overall average root mean square (RMS) EMG amplitude from the steps without, during and after SP and/or PT transspinal stimulation delivered across all 16 bins of the step cycle. Asterisks indicate significant differences during SP and PT transspinal stimulation compared to the remaining stepping conditions, supporting for locomotor EMG recovery at the steps after SP or PT transspinal stimulation. Plus sign indicates significant difference during SP and PT transspinal stimulation.

In control subjects, the SOL, MG, PL, and TA EMG activity from both legs was decreased in amplitude at the steps during transspinal stimulation when compared to the steps without stimulation (Figure 2A). The EMG depression was of similar strength for steps during single pulse or pulse train transspinal stimulation (Figure 2A). The EMG activity of all muscles from both legs recovered fully at the step immediately after a single pulse or pulse train transspinal stimulation (Figure 2A). Because the EMG patterns at steps during and after a single pulse or pulse train transspinal stimulation were strongly correlated to the EMG patterns at steps without stimulation (Spearman’s rank correlation; *r_s_* = 0.684–0.961, *p* < 0.001; Table 2), we can theorize that transspinal stimulation decreases locomotor EMG amplitude but does not affect the phase-dependent EMG modulation pattern in control subjects (Figure 2A).

**Table 2:** Spearman’s rank correlation coefficients for electromyographic (EMG) activity without, during, and after transspinal stimulation.

		EMG activity for steps during and after stimulation compared to the EMG activity at steps without stimulation			
	Recorded muscle	Single-pulse	Pulse train	After single pulse	Afterpulse train
Able-bodied	L SOL	0.802	0.852	0.917	0.923
	L MG	0.838	0.811	0.904	0.896
	L PL	0.684	0.695	0.946	0.956
	L TA	0.815	0.832	0.935	0.918
	R SOL	0.850	0.899	0.948	0.919
	R MG	0.829	0.820	0.903	0.857
	R PL	0.886	0.801	0.947	0.961
	R TA	0.738	0.817	0.912	0.893
SCI	L SOL	−0.202	−0.191	0.865	0.912
	L MG	0.013	−0.148	0.883	0.922
	L PL	−0.628	−0.494	0.629	0.620
	L TA	−0.203	−0.106	−0.015	−0.110
	R SOL	0.581	0.324	0.804	0.786
	R MG	−0.556	−0.444	0.836	0.821
	R PL	0.422	0.562	0.834	0.898
	R TA	0.064	−0.183	0.828	0.857

*The Spearman’s rank correlation was established between the EMG activity pattern observed across the whole duration between the steps without stimulation and during and/or after a single pulse or pulse train transspinal stimulation. A negative value suggests an opposite association between the two signals and thus reversal of EMG modulation pattern. SCI, spinal cord injury; L, left; R, right; SOL, soleus; MG, medial gastrocnemius; PL, peroneus longus; TA, tibialis anterior*.

The RMS EMG amplitude was significantly different in the steps without, during single pulse or pulse train, and after transspinal stimulation for the left SOL (*F*_(4,48)_ = 48.3, *p* < 0.001), MG (*F*_(4,48)_ = 47.2, *p* < 0.001), PL (*F*_(4,48)_ = 78.4, *p* < 0.001) and TA (*F*_(4,48)_ = 58.94, *p* < 0.001) muscles (Figure 2B). Similar results were found for the right SOL (*F*_(4,48)_ = 83.3, *p* < 0.001), MG (*F*_(4,48)_ = 57.5, *p* < 0.001), PL (*F*_(4,48)_ = 26.8, *p* < 0.001), and TA (*F*_(4,48)_ = 90.5, *p* < 0.001) muscles (Figure 2B). Pairwise Bonferroni post-hoc t-tests showed that the RMS EMG amplitude of all muscles at the steps during single pulse or pulse train stimulation was equally reduced compared to the steps without or after stimulation (for both *p* < 0.05).

**Figure 3:**
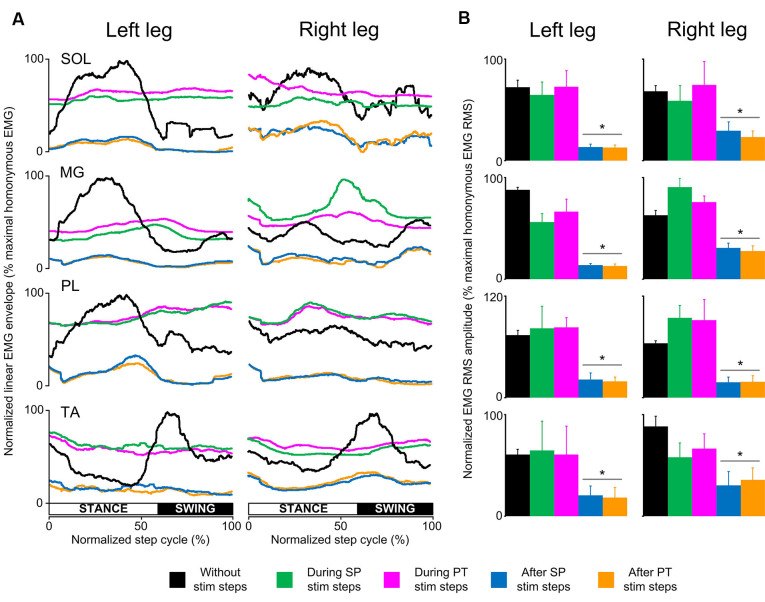
Locomotor EMG activity without, during, and after transspinal stimulation in spinal cord injury (SCI) subjects. **(A)** Overall amplitude of normalized locomotor electromyographic (EMG) activity from soleus (SOL), medial gastrocnemius (MG), peroneus longus (PL), and tibialis anterior (TA) muscles during walking with the Lokomat6^®^Pro without and during the single pulse (SP; 1 ms pulse width) or pulse train (PT; 12 pulses of 33 ms total duration) transspinal stimulation. **(B)** Overall average root mean square (RMS) EMG amplitude from the steps without, during, and after SP and PT transspinal stimulation delivered across all 16 bins of the step cycle. Asterisks indicate significant differences after SP and PT transspinal stimulation compared to the remaining stepping conditions, supporting the inability of spinal locomotor centers to overcome the simultaneous depolarization of multiple motoneurons following transspinal stimulation in SCI subjects.

In SCI subjects, the EMG activity was depressed only at the steps immediately after a single pulse or pulse train transspinal stimulation (Figure 3A). Single-pulse or pulse train transspinal stimulation disrupted the already pathological EMG modulation pattern present at the steps without stimulation (Figure 3A). We observed a negative or weak Spearman’s rank correlation between the EMG activity for steps with and without transspinal stimulation (Table 2), supporting further the above findings. The EMG activity from the steps immediately after transspinal stimulation showed a strong association to that observed at steps without stimulation (*r_s_* = 0.629–0.921, *p* < 0.001; Table 2).

The RMS EMG amplitude was significantly different in the steps without, during single pulse or pulse train and after transspinal stimulation for the left SOL (*F*_(4,16)_ = 16.1, *p* < 0.001), MG (*F*_(4,16)_ = 33.6, *p* < 0.001), PL (*F*_(4,16)_ = 7.44, *p* = 0.001), and TA (*F*_(4,16)_ = 4.5, *p* = 0.03) muscles (Figure 3B). Similar results were found for the right SOL (*F*_(4,16)_ = 6.99, *p* = 0.002), MG (*F*_(4,16)_ = 11.4, *p* < 0.001), PL (*F*_(4,16)_ = 9.87, *p* < 0.001) and TA (*F*_(4,16)_ = 14.6, *p* < 0.001) muscles (Figure 3B). Pairwise Bonferroni post-hoc t-tests showed that the RMS EMG amplitude at the steps immediately after single pulse or pulse train transspinal stimulation was significantly reduced compared to the RMS EMG obtained at the steps without and during stimulation for all muscles (p < 0.05; Figure 3B).

### Mean Power Frequency of Locomotor EMG Activity During Transspinal Stimulation

**Figure 4:**
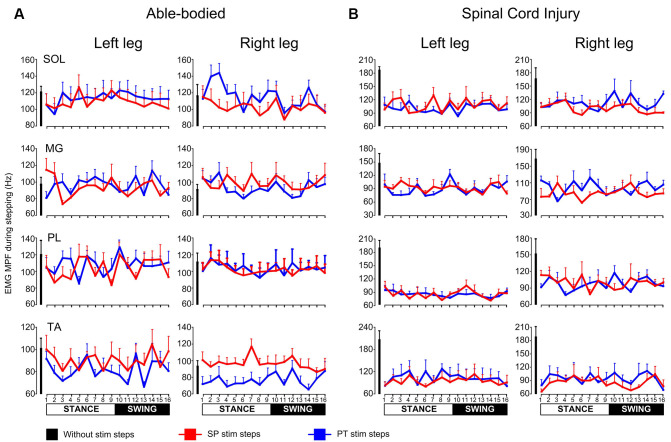
Mean power frequency (MPF) of electromyographic (EMG) power spectrum during walking. The overall MPF (Hz) from left and right soleus (SOL), medial gastrocnemius (MG), peroneus longus (PL), and tibialis anterior (TA) muscles during walking on a motorized treadmill in able bodied subjects **(A)** or with the Lokomat6^®^Pro in individuals with spinal cord injury (SCI; **B**). The MPF is shown for the steps without or during single pulse (SP) or pulse train (PT) transspinal stimulation at each bin of the step cycle. The step cycle was divided into 16 equal bins. Bin 1 corresponds to heel contact. Bins 8, 9, and 16 correspond approximately to stance-to-swing transition, swing phase initiation, and swing-to-stance transition, respectively.

In control subjects, the MPF was similar for steps during single pulse or pulse train transspinal stimulation compared to that observed for steps without stimulation at all bins for the left SOL (H_(32)_ = 15.25, *p* = 0.99), MG (H_(32)_ = 37.97, *p* = 0.20), PL (H_(32)_ = 35.72, *p* = 0.29), TA (H_(32)_ = 30.71; *p* = 0.53) and the right SOL (H_(32)_ = 37.62; *p* = 0.22), MG (H_(32)_ = 23.95; *p* = 0.84), right PL (H_(32)_ = 6.74; *p* = 1.00), but not for the right TA (H_(32)_ = 70.71; *p* = 0.001) muscles (Figure 4A). In SCI subjects, the MPF was similar for steps during single pulse or pulse train transspinal stimulation compared to that observed for steps without stimulation for all muscles and step cycle phases (Figure 4B), except for the right MG that the MPF was decreased at bin 3 during pulse train and at bin 6 during single pulse transspinal stimulation compared to the MPF observed without stimulation (left/right SOL: H_(32)_ = 30.79, *p* = 0.52; H_(32)_ = 32.83, *p* = 0.42, left/right MG: H_(32)_ = 30.15, *p* = 0.56; H_(32)_ = 53.66, *p* = 0.01, left/right PL: H_(32)_ = 31.08, *p* = 0.51; H_(32)_ = 21.2, *p* = 0.92, left/right TA: H_(32)_ = 25.06, *p* = 0.80; H_(32)_ = 42.95, *p* = 0.09).

### Intralimb and Interlimb Coordination During Transspinal Stimulation

**Figure 5:**
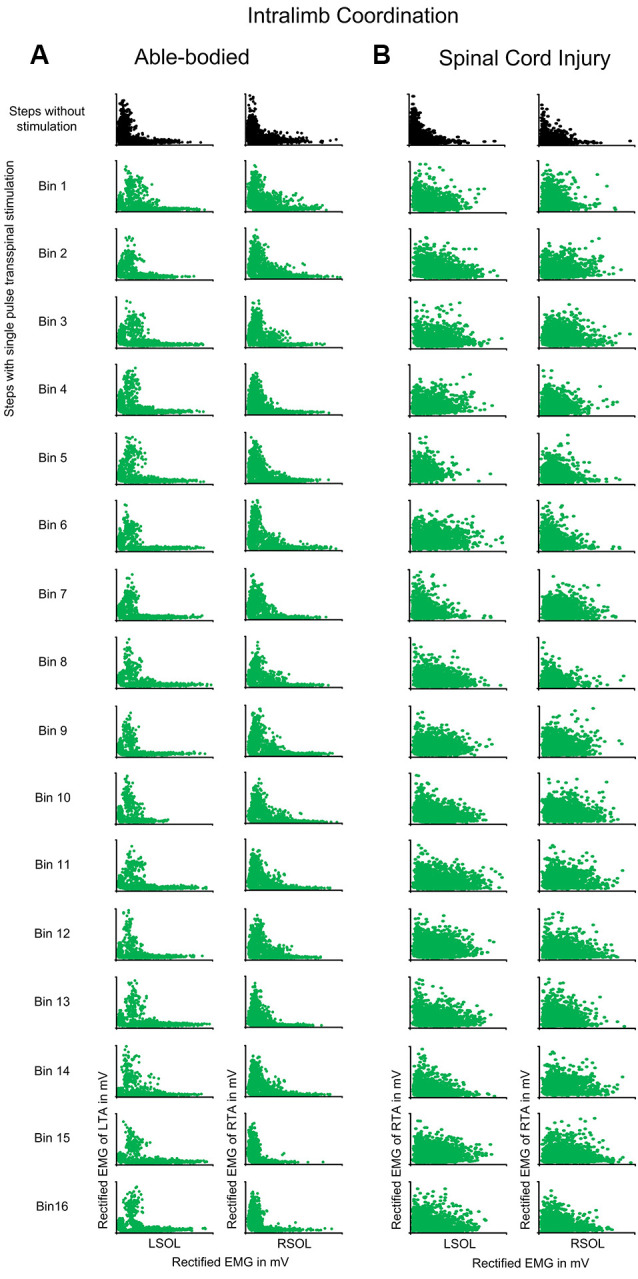
Intralimb coordination during transspinal stimulation. The electromyographic (EMG) activity of the soleus (SOL) muscle is plotted against that of the antagonistic tibialis anterior (TA) muscle for the right and left legs, and for the steps without (top row: black) and during single pulse transspinal stimulation delivered randomly at each bin of the step cycle (green) in able-bodied subjects **(A)** and individuals with spinal cord injury (SCI) **(B)**. An *L*-shape supports physiological intralimb coordination and alternated activity between the antagonistic SOL and TA muscles. No changes in the *L*-shape were observed in able-bodied subjects **(A)**. In contrast, single pulse transspinal stimulation distorted the *L*-shape, and thus intralimb coordination in SCI subjects **(B)**. The step cycle was divided into 16 equal bins. Bin 1 corresponds to heel contact. Bins 8, 9, and 16 correspond approximately to stance-to-swing transition, swing phase initiation, and swing-to-stance transition, respectively.

In control subjects, intralimb coordination was not affected when single-pulse transspinal stimulation was delivered randomly at different phases of the step cycle (Figure 5A). The same results were observed with pulse train transspinal stimulation but are not shown in graphs. A 2-sample KS test confirmed that the EMG activity distribution shape during stimulation remained similar to that observed at steps without stimulation in both left and right legs (Figure 5A). In Table 3, the D-stat values of the EMG shape distribution at steps without and during the single pulse transspinal stimulation delivered in each bin of the step cycle are indicated. For example, at the mid-stance phase (bin 5) the difference in D-stat value was 3.9% in the left SOL/TA, and 1.15% for the right SOL/TA for steps with and without stimulation, respectively (Table 3). Because these D-stat values differed by a probability value of less than 5% (Lehmann and D’Abrera, 1975), we can suggest that intralimb coordination for steps with and without stimulation was not significantly different in control subjects. This result was found for all bins of the step cycle (Figure 5A and Table 3). In SCI subjects, the weak intralimb coordination for the steps during single pulse transspinal stimulation compared to that observed without stimulation (Figure 5B) support for co-contraction between ankle antagonistic muscles. For example, at bin 5 the difference in D-stat values was 23% for the left and 11% for the right EMG coordination between the steps with and without stimulation (Table 3).

**Table 3:** Two-sample Kolmogorov-Smirnov probability values of intralimb and interlimb coordination for steps with and without stimulation.


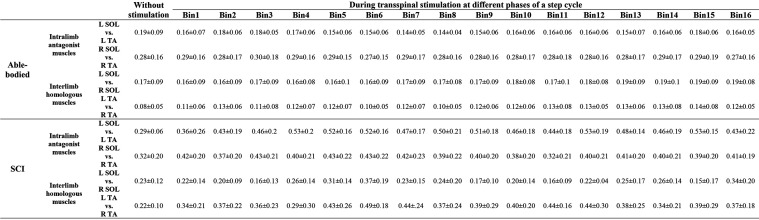

*Two-sample Kolmogorov-Smirnov test values between the two electromyographic (EMG) data sets observed separately for steps without stimulation and during single pulse transspinal stimulation at each bin of the step cycle are indicated between homonymous muscles of both legs or antagonistic muscles of one leg underlying interlimb and intralimb coordination. In control subjects, the small difference (less than 5%) in values observed without transspinal stimulation compared to those during stimulation suggest that the shape of the distribution between two EMG signals is similar. In spinal cord injury (SCI) subjects, the values were different (more than 5%), supporting for altered intralimb and interlimb coordination during transpinal stimulation. Values are mean ± SD. SOL, soleus; MG, medial gastrocnemius; PL, peroneus longus; TA, tibialis anterior; L, left; R, right*.

**Figure 6:**
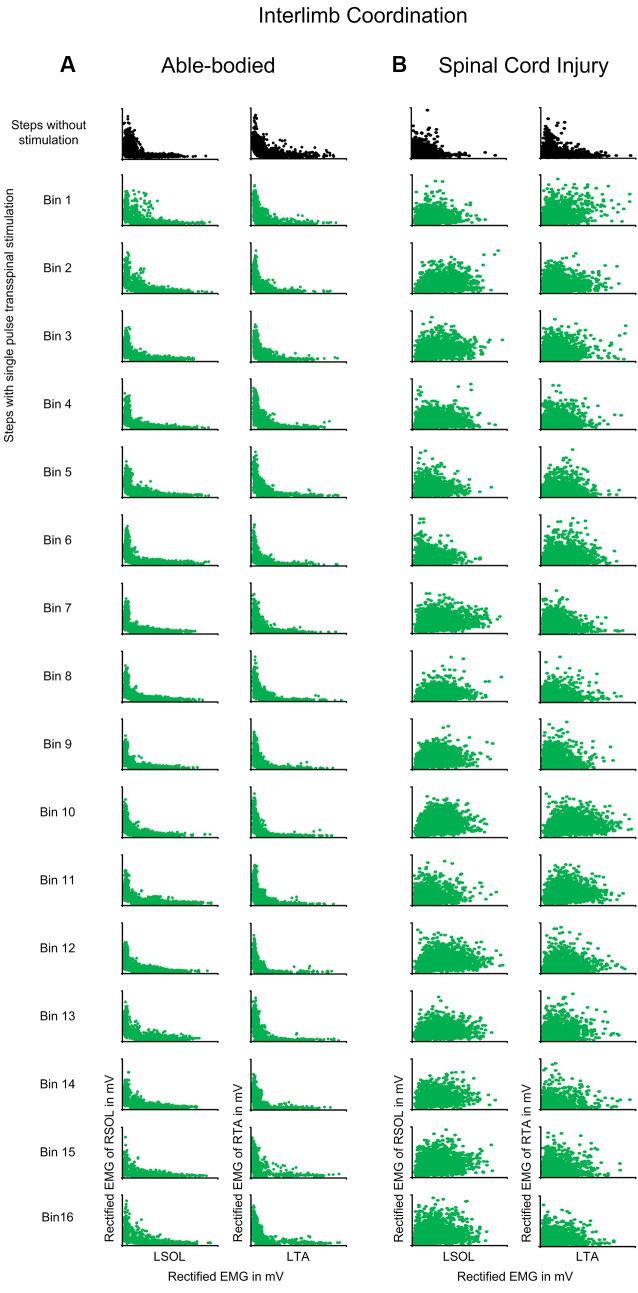
Interlimb coordination during transspinal stimulation. The electromyographic (EMG) activity of the right soleus (SOL) or tibialis anterior (TA) muscles is plotted against that of the left homologous muscle (SOL or TA) for steps without (top row: black) and during single pulse transspinal stimulation delivered randomly at each bin of the step cycle (green) in able-bodied subjects and individuals with spinal cord injury (SCI). An *L*-shape supports reciprocal activity between the homologous SOL or TA muscles and thus interlimb coordination. No changes in the *L*-shape were observed in able-bodied subjects **(A)**. In contrast, single pulse transspinal stimulation distorted the *L*-shape, and thus interlimb coordination in SCI subjects **(B)**. The step cycle was divided into 16 equal bins. Bin 1 corresponds to heel contact. Bins 8, 9, and 16 correspond approximately to stance-to-swing transition, swing phase initiation, and swing-to-stance transition, respectively.

Interlimb coordination was not affected in control subjects when single-pulse transspinal stimulation was delivered randomly at different phases of the step cycle (Figure 6A). The D-stat values were different by less than 5% for steps with and without stimulation (Table 3). In SCI subjects, transspinal stimulation augmented the otherwise pathological interlimb coordination by increasing further the co-contraction between the left and right SOL and TA muscles (Figure 6B). For example, at bin 5 the difference in D-stat values was 8% (left vs. right SOL) and 21% (left vs. right TA) between the steps without and with stimulation (Table 3).

### Modulatory Effects of Transspinal Stimulation on Soleus H-Reflex Amplitude During Stepping in Humans With and Without SCI

**Figure 7:**
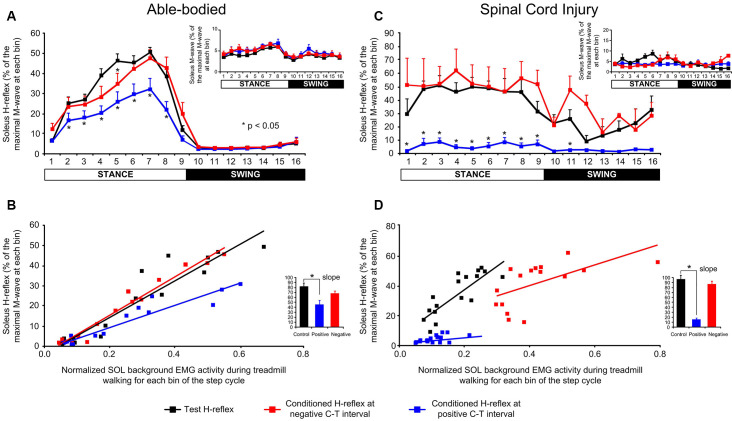
Soleus H-reflex amplitude modulation following a transpinal stimulation during walking. **(A–C)** Overall soleus H-reflex amplitude at each bin of the step cycle recorded under control conditions and following single-pulse transspinal conditioning stimulation at a negative or positive conditioning-test (C-T) interval in able-bodied and spinal cord injury (SCI) subjects. The soleus H-reflex is shown as a percentage of the maximal M-wave evoked 60 ms after the test pulse. The amplitude of the soleus M-waves for each bin of the step cycle is also shown. For both subject groups, the group mean amplitude of soleus H-reflexes and M-waves are shown. **(B–D)** The linear relationship between soleus background electromyographic (EMG) activity and normalized soleus H-reflex amplitude in able-bodied and SCI subjects. For both subject groups, the overall amplitude of the slope is also indicated. Asterisks indicate significant differences between the control and conditioned H-reflex. The step cycle was divided into 16 equal bins. Bin 1 corresponds to heel contact. Bins 8, 9, and 16 correspond approximately to stance-to-swing transition, swing phase initiation, and swing-to-stance transition, respectively.

In control subjects, transspinal stimulation produced significant changes across the different phases of the step cycle (*F*_(15,537)_ = 73.28, *p* < 0.001) and conditioning stimulation (*F*_(2,537)_ = 28.031, *p* < 0.001), while a significant interaction between bins and type of H-reflex was found (*F*_(30,537)_ = 2.468, *p* < 0.001). Specifically, Holm-Sidak multiple comparisons showed that transspinal stimulation at a positive C-T interval depressed the soleus H-reflex throughout the stance phase (bins 2–8; *p* < 0.001) compared to the control H-reflexes. At the negative C-T interval, soleus H-reflex depression was present only at bin 5 (*t* = 2.54, *p* = 0.011; Figure 7A). Please note that at the negative C-T interval the TEP was not subtracted from the H-reflex which is known that these compound action potentials summate in the surface EMG. The soleus H-reflex excitability changes during stepping occurred with constant M-waves (*F*_(2,537)_ = 4.82, *p* = 0.08; Figure 7A), supporting stable recording and stimulation procedures. The soleus H-reflex depression at the positive C-T interval coincided with decreased slope (*F*_(2, 34)_ = 4.65, *p* = 0.016; Figure 7B) of the linear relationship between the background EMG activity and H-reflex.

In SCI subjects, transspinal stimulation produced significant changes across the different phases of the step cycle (*F*_(15,176)_ = 6.86, *p* < 0.001) and conditioning stimulation (*F*_(2,176)_ = 156.8, *p* < 0.001), while a significant interaction between bins and type of H-reflex was found (*F*_(30,176)_ = 1.85, *p* = 0.007). Specifically, Bonferroni t-tests showed that transspinal stimulation at a positive C-T interval reduced the soleus H-reflex amplitude at bins 2–9 of the step cycle compared to control H-reflexes or following transspinal conditioning stimulation at a negative C-T interval (Figure 7C). M-waves were constant under control and conditioning reflex recordings (*F*_(2,176)_ = 8.23, *p* = 0.05; Figure 7C) during robotic-assisted stepping. The H-reflex gain obtained from the slope of the linear relationship between the background EMG activity and H-reflex was significantly decreased in the positive transspinal stimulation protocol (Figure 7D).

## Discussion

We have provided evidence on the actions of transspinal stimulation on locomotor networks in able-bodied and SCI individuals. Transspinal stimulation, when delivered before posterior tibial nerve stimulation, reduced H-reflex excitability throughout the step cycle with near-complete H-reflex depression in individuals with SCI. Moreover, transspinal stimulation had differential effects on locomotor muscle activity in able-bodied and SCI individuals. In fact, locomotor muscle activity in able-bodied individuals was greatly reduced but retained its phase-dependent modulation pattern during transspinal stimulation. In contrast, the phase-dependent locomotor muscle activity in SCI individuals was replaced with tonic activity throughout the step cycle. The injured human spinal cord, therefore, may not be able to counteract the complex neuronal phenomena accompanying transspinal stimulation at suprathreshold intensities. Spinal motoneurons after SCI are at an increased excitability state as a result of changes in their physiological and biophysical properties (Nielsen et al., 2007; D’Amico et al., 2014). These changes include but are not limited to depolarized resting membrane potentials, hyperpolarized spike thresholds, shortened after-hyperpolarization duration, and enhanced formation of both Ca^2+^ and Na^+^-mediated persistent inward currents (Bennett et al., 2001; Gorassini et al., 2004; Li et al., 2004). The altered intrinsic properties of motoneurons contribute partly to reflex hyperexcitability and spasticity (Nielsen et al., 2007; D’Amico et al., 2014), with interventions controlling pathological expressions of muscle tone to be in great need.

Potential targets of transspinal stimulation include proprioceptive afferents and spinal and brain neural circuits *via* dorsal column axons (Costa and Deletis, 2016). Transspinal stimulation activates neuronal pathways that convey both descending motor drive and ascending sensory inputs (Knikou and Murray, 2018; Murray et al., 2019). Specifically, MEPs and spinally-induced TEPs summate at the surface EMG when transcortical stimulation is delivered ~10 ms before transspinal stimulation (Knikou, 2014). Moreover, transspinal stimulation decreases corticospinal excitability during walking likely *via* depression of indirect descending waves (Takeoka et al., 2014; Pulverenti et al., 2019), and modulates the afferent-mediated MEP facilitation (Knikou et al., 2015). Given the similar neuronal interactions between the soleus H-reflex and the soleus TEP (Knikou and Murray, 2018), transspinal stimulation induces neuromodulation across broad cortical, corticospinal, and spinal neural networks. In this study, we observed distinct differences in the timing of locomotor EMG depression at the steps during and immediately after a single pulse or brief pulse train transspinal stimulation in able-bodied and SCI individuals (Figures 2, 3). The difference among able-bodied and SCI subjects may be the result of pathological activation of supraspinal neuronal pathways by transspinal stimulation and pathological integration of descending motor drive with spinal neural circuits in SCI subjects (Ellaway et al., 2007). Additionally, the inability of spinal locomotor networks to counteract the simultaneous depolarization of multiple motoneurons over several spinal segments produced by transspinal stimulation in SCI subjects might contribute to the different EMG amplitude modulation.

In addition to supraspinal-mediated effects, transspinal stimulation could have affected the activity of local spinal inhibitory neuronal networks including but not limited to recurrent and reciprocal inhibitory circuits (Maruyama et al., 1982; Maertens de Noordhout et al., 1988; Hunter and Ashby, 1994; Gaunt et al., 2006; Ladenbauer et al., 2010; Sharpe and Jackson, 2014). Subsequently, the pathological behavior of spinal inhibitory neuronal networks in SCI may have caused the near-constant activity in locomotor EMG during transspinal stimulation (Figure 3). In contrast, the physiological spinal inhibition in able-bodied individuals is adjusted immediately at the step after transspinal stimulation bringing the EMG activity to the level observed without stimulation. At this point, we should consider that similar mechanisms or neuronal pathways are involved in the generalized depression of EMG activity during walking following single pulse and pulse train transspinal stimulation. This is supported by similar modulatory actions although the stimulation intensity was greatly different in the single pulse and pulse train transspinal stimulation protocols. While only single pulse and not pulse train transspinal stimulation produced synchronized depolarization of flexor and extensor motoneurons that is demonstrated in the surface EMG as TEPs, both can potentially excite cutaneous afferents that in turn are known to affect the activity of spinal interneurons that modulate depolarization of motoneurones *via* presynaptic and postsynaptic inhibition (Knikou, 2008; Côté et al., 2018). This is supported by the recent findings on inhibition of locomotor-like activity and altered gain of cutaneous reflexes in spinal cats following mechanical stimulation of the spinal cord (Merlet et al., 2020).

The unchanged MPF (Figure 4) supports further that the effects were not due to peripheral sources such as alterations in motor unit firing rate. Because the basic locomotor EMG pattern was preserved, we can theorize that transspinal stimulation perturbs the outputs of common stepping generator(s) that control leg muscle activity. The EMG and soleus H-reflex depression in humans with and without SCI support for homologous neural mechanisms mediating reduced reflex excitability and motor output.

Transspinal stimulation exacerbated the already impaired intralimb and interlimb coordination in SCI individuals (Figures 5, 6). Intralimb and interlimb coordination require the physiological function of interneurons that are considered part of the central pattern generator (McCrea and Rybak, 2007; Kiehn, 2016; Pocratsky et al., 2017). These interneurons include group Ia and commissural interneurons that project to several different classes of inhibitory interneurons like Renshaw cells and Ia inhibitory interneurons, and act also by direct monosynaptic excitation and/or inhibition to motoneurons (Côté et al., 2018). When compared to findings in control subjects, the EMG phase-dependent modulation at the steps during transspinal stimulation was similar to that observed without transspinal stimulation, while both intralimb and interlimb coordination remained unchanged (Figure 4). Consequently, we conclude that the impaired intralimb and interlimb coordination, amplitude of motoneuronal depolarization, and phasic activity of motoneurons in SCI individuals were likely the result of the already malfunctioned spinal locomotor networks and their inability to counteract the strong tonic excitatory inputs following transspinal stimulation in SCI individuals.

In SCI individuals, a near-full depression of the soleus H-reflex throughout the step cycle was evident (Figure 7C). While the soleus background EMG level was small at the positive conditioning transspinal protocol, the soleus H-reflex amplitude modulation during walking is not a mere reflection of background EMG activity because the H-reflex amplitude during locomotion does not depend on the excitation level of motoneurons (Capaday and Stein, 1986, 1987a; Dietz et al., 1990; Ferris et al., 2001). The reduced activity of ankle extensor muscles coincided with soleus H-reflex depression during the stance phase of walking in able-bodied subjects while the phase-dependent amplitude modulation was maintained (Figure 7A). The reduced reflex gain and altered threshold in SCI individuals suggest possible involvement of increases in threshold *via* GABA receptors by transspinal stimulation (Ferris et al., 2001). These are not unexpected results because group Ia pathways contribute significantly to ankle extensor EMG activity during walking (Mazzaro et al., 2005). Our findings are consistent with those we recently reported on the time course of transspinal conditioning stimulation effects on the soleus H-reflex in resting healthy individuals (Knikou and Murray, 2018). The soleus H-reflex depression in the present study may be caused by two mechanisms: (1) collision between TEPs and H-reflexes in peripheral sensory axons; and/or (2) transspinal stimulation-induced activity of inhibitory interneurons acting pre- or post-synaptically on the Ia-motoneuron synapse. At short-positive C-T intervals, transspinal stimulation-evoked TEPs travel both ortho- and antidromically in excited afferents (Hunter and Ashby, 1994; Buonocore et al., 2008). Thus, at short-positive C-T intervals, orthodromically traveling Ia afferent volleys may have collided with antidromically traveling TEP volleys (Formento et al., 2018). On the other hand, at the positive C-T intervals, transspinal stimulation has ample time to excite directly or indirectly spinal interneuronal circuits through action potentials traveling rostrally on dorsal roots and caudally on ventral roots (Yiannikas and Shahani, 1988) depolarizing Ia afferent terminals and thus initiating interneuronal activity (Wall, 1958) resulting in decreased spinal reflex excitability during the stance phase of walking (Knikou, 2008; Côté et al., 2018). Moreover, transspinal stimulation innervates dorsal roots that also transynaptically excite anterior rootlets through the activation of spinal interneurons, which may interfere with the reflex arcs and hence to the production of H-reflex (Yiannikas and Shahani, 1988; Hofstoetter et al., 2014).

When transspinal stimulation is delivered after tibial nerve stimulation (i.e., negative C-T interval), the soleus alpha motoneurons are depolarized monosynaptically by muscle spindle group Ia afferent volleys before being activated by transspinal stimulation, while both TEP and H-reflex summate in the surface EMG (Figure 1), as we have previously documented (Knikou and Murray, 2018). The absent soleus H-reflex depression at the negative C-T interval in both subject groups support further the possibility of TEP and Ia afferent volley collisions and/or involvement of spinal inhibitory interneuronal circuits at the positive C-T interval. Based on the observed effects, we conclude that transspinal stimulation at intensities that evoke responses in the leg muscles decreases soleus H-reflex in SCI individuals, and thus may be used to control hyperreflexia during walking. The reflexively-mediated control of locomotion and reflexively-mediated recovery of locomotion after SCI (Knikou, 2010; Dietz and Fouad, 2014) supports the clinical importance of delivering transspinal stimulation at suprathreshold intensities. On the other hand, suprathreshold transspinal stimulation reduced, or nearly occluded, Ia afferent volleys (i.e., soleus H-reflex) during walking which may have contributed to the impairment in interlimb and intralimb coordination. Consequently, this effect may have negative consequences on the rehabilitation potential of transspinal combined with locomotor training (Akay et al., 2014; Takeoka et al., 2014; Takeoka and Arber, 2019). Subthreshold intensities may change the excitability state of the spinal cord (Minassian et al., 2007; Hofstoetter et al., 2017) without the detrimental effects we observed in the current study on limb coordination and muscle activity, but further research is needed to establish the neurophysiological changes upon subthreshold transspinal stimulation.

At this point, we should note that the conditioning effects we report in this study are not comparable to those when transspinal stimulation is used as a rehabilitation intervention in humans after SCI. This is because tonic stimulation may have different effects compared to a conditioning experimental protocol. For example, multiple sessions of tonic low frequency (0.2 Hz) transspinal stimulation increases motoneuron output and simultaneously decreases reflex excitability and clinically evaluated hyperreflexia in both, motor-complete and incomplete SCI, while transspinal stimulation at medium frequencies (15–50 Hz) generates locomotor-like motor patterns in cases of minimal descending motor control and modifies spasticity (Shapkova and Schomburg, 2001; Minassian et al., 2007; Gorodnichev et al., 2012; Hofstoetter et al., 2015, 2017; Knikou and Murray, 2019; Murray and Knikou, 2019). It is worth noting that changes in amplitude of locomotor EMG activity remain to be shown when transspinal stimulation is delivered during daily sessions of assisted stepping. In this case, however real-time activation of the left and right neural circuits of locomotion independently during stepping are needed so to activate spinal interneuronal networks physiologically. This arrangement will counteract the fact that transspinal stimulation did not selectively target the left or the right half of the spinal cord with respect to the phase of the step cycle of the corresponding leg in our current study (Wagner et al., 2018; Calvert et al., 2019).

### Limitations of the Study

There are, however, limitations of the current study that should be considered. The demographics of the SCI population are largely heteronymous with large variation in injury severity (AIS scores B-D), years post-injury, and participant ages. Therefore, further experiments are needed with a larger population of individuals with SCI to more robustly define the effects of transspinal stimulation on H-reflex modulation during stepping and to stratify based on more homogenous groups of individuals with SCI. Last, while depression of reflex excitability can have significant clinical benefits, behavioral, clinical, and side effects remain to be established *via* randomized clinical trials in individuals with SCI.

### Conclusion

We demonstrated that transspinal stimulation at suprathreshold intensities with a single pulse or brief pulse train decreased the amplitude of locomotor EMG activity and impaired interlimb and intralimb coordination in SCI individuals. In healthy subjects, the EMG activity was decreased at the steps during but not immediately after transspinal stimulation. Transspinal stimulation produced depression on the soleus H-reflex during walking which was stronger in SCI individuals. The similarities of the observed effects in people with and without SCI strongly support the notion that transspinal stimulation accesses spinal locomotor networks. Further studies are required to determine the effects of transspinal stimulation when delivered during assisted stepping on locomotor function in individuals with SCI.

## Data Availability Statement

The original contributions presented in the study are included in the article, further inquiries can be directed to the corresponding author.

## Ethics Statement

The studies involving human participants were reviewed and approved by City University of New York Institutional Review Board Committee. The patients/participants provided their written informed consent to participate in this study.

## Author Contributions

MK: conception and design of research, interpreted results of experiments and wrote the first draft of the manuscript. MI, TP, and MK: performed experiments. MI, MK and analyzed data. MI, and MK prepared figures. MI, TP, and MK: edited and revised manuscript. MI, TP, and MK: approved the final version of the manuscript. All authors contributed to the article and approved the submitted version.

## Conflict of Interest

The authors declare that the research was conducted in the absence of any commercial or financial relationships that could be construed as a potential conflict of interest.
